# Strengths and Weaknesses of Pre-Clinical Models for Human Melanoma Treatment: Dawn of Dogs’ Revolution for Immunotherapy

**DOI:** 10.3390/ijms19030799

**Published:** 2018-03-10

**Authors:** Giuseppina Barutello, Valeria Rolih, Maddalena Arigoni, Lidia Tarone, Laura Conti, Elena Quaglino, Paolo Buracco, Federica Cavallo, Federica Riccardo

**Affiliations:** 1 Department of Molecular Biotechnology and Health Sciences, University of Torino, 10126 Torino, Italy; giuseppina.barutello@unito.it (G.B.); valeria.rolih@unito.it (V.R.); maddalena.arigoni@unito.it (M.A.); lidia.tarone@edu.unito.it (L.T.); laura.conti@unito.it (L.C.); elena.quaglino@unito.it (E.Q.); federica.cavallo@unito.it (F.C.); 2 Department of Veterinary Science, University of Torino, 10095 Grugliasco, Italy; paolo.buracco@unito.it

**Keywords:** melanoma, pre-clinical models, immunotherapy, canine melanoma, comparative oncology, CSPG4

## Abstract

Despite several therapeutic advances, malignant melanoma still remains a fatal disease for which novel and long-term curative treatments are needed. The successful development of innovative therapies strongly depends on the availability of appropriate pre-clinical models. For this purpose, several mouse models holding the promise to provide insight into molecular biology and clinical behavior of melanoma have been generated. The most relevant ones and their contribution for the advancement of therapeutic approaches for the treatment of human melanoma patients will be here summarized. However, as models, mice do not recapitulate all the features of human melanoma, thus their strengths and weaknesses need to be carefully identified and considered for the translation of the results into the human clinics. In this panorama, the concept of comparative oncology acquires a priceless value. The revolutionary importance of spontaneous canine melanoma as a translational model for the pre-clinical investigation of melanoma progression and treatment will be here discussed, with a special consideration to the development of innovative immunotherapeutic approaches.

## 1. Introduction

Melanoma is the sixth most common leading cancer worldwide and its incidence is continuously rising [1]. While in its early stages melanoma is highly curable, the management of metastatic melanoma patients has been historically exceptionally challenging, with extremely poor survival rate [2,3]. Thus, the identification of innovative and long-term curative treatments is still an unmet need. For this purpose, researchers have looked for models that could represent melanoma development and progression in a realistic way.

For many years, the study of melanoma cell biology and anti-melanoma drug activity has been performed exploiting cultured tumor cell lines in vitro. However, this model is not suitable for an accurate reproduction of melanoma complexity, lacking several fundamental aspects. Growing in a two-dimensional setting, in vitro cell lines do not mimic the three-dimensional architecture of a melanoma, neither its heterogeneity or its dynamic connection with the microenvironment. Indeed, during the natural evolution of the tumor, melanoma cells establish complex interactions with the surrounding cells, including immune ones, which play a fundamental role in shaping melanoma evolution [4,5].

The investigation of in vivo pre-clinical models could provide a valuable tool for gaining insight into melanoma development, progression and clinical behavior in a more realistic way which retains most of the principal hallmarks of cancer [6,7,8]. In this panorama, mice developing melanoma have been generated and widely explored. Some pre-clinical studies in mouse models have been of paramount importance for the human clinics, but several failed in the translation from bench to bedside [9]. Spontaneous canine melanoma could represent a revolution in the field, allowing to overcome the limitations of pre-clinical mouse models. Moreover, the evaluation of therapeutic approaches to fight against melanoma in dogs represents a valuable option to accelerate the translation of pre-clinical studies from the veterinary to the human clinical oncology, with the benefit for both species [7]. The principal pre-clinical mouse models and their contribution in the advancement in therapeutic approaches for human melanoma treatment will be here summarized. A special focus will be dedicated to the emerging importance of immunotherapeutic studies performed in canine melanoma patients.

## 2. The Strength of Mouse Models: Testing of Targeted Therapies against Melanoma

Mouse models of melanoma have come to the stage, providing instrumental information on this complex disease. They have allowed a better understanding of melanoma pathobiology through the (i) identification and characterization of specific key tumor suppressor genes and oncogenes involved in melanoma biology; (ii) the testing of novel therapeutic agents to be applied in the treatment of melanoma patients and (iii) the elucidation of the role of circulating exosomes (EXs) as new predictors or early markers of metastatic disease.

By definition, models will not replicate all aspects of human melanoma. However, in multiple settings, mice really served as little patients, successfully linking bench to bedside. In the following sections, mouse models used to reproduce the more relevant genetic aberrations identified in human melanomas will be discussed.

The genetic characterization of melanoma is still incomplete. Nevertheless, molecular analysis of both familial and sporadic cutaneous melanomas has led to the identification of recurrent somatic mutations in several key genes relevant for cell proliferation (BRAF, NRAS and NF1), growth and metabolism (PTEN and KIT), resistance to apoptosis (TP53), cell cycle control (CDKN2A) and replicative lifespan (TERT) [10]. A different mutational pattern has been highlighted in melanomas arisen from other tissues, including the uvea of the eye and within mucous membranes, characterized by frequent mutations in the guanine-nucleotide binding protein G subunit alpha Q (GNAQ) and alpha 11 (GNA11), in the tumor suppressor gene BRCA1 associated protein 1 (BAP1) or in splicing factor 3b subunit 1 (SF3B1). Most of these genetic aberrations have been reproduced in mouse models, in order to better elucidate their functions in melanoma progression and their role as potential therapeutic targets [11].

### 2.1. Pre-Clinical Modeling of BRAF in Mice

In 2002, for the first time, Davies and collaborators reported the presence of the—now well-known—somatic point mutation V600E in the BRAF gene in human malignant melanomas [12]. It was the beginning of accumulating investigations that validated the expression of BRAF^V600E^ in over 50% of human melanoma patients, suggesting its causal role in melanoma initiation, development, and progression [13,14,15,16]. It was the starting of hope for a potential revolution in the management of the majority of melanoma patients thanks to the exploration of target-specific therapies directed against BRAF^V600E^. As a straight consequence, a number of murine models were developed, including genetically engineered mouse (GEM) and human xenograft models which independently proved the oncogenic role and importance of BRAF^V600E^ mutation in melanomagenesis.

In particular, in 2009, Dhomen and collaborators developed a GEM model (GEMM) of BRAF^V600E^ melanoma (Tyr::CreERT2, BRAF^V600E^), whose tumors recapitulate human amelanotic/oligomelanotic malignant melanoma. This GEMM was extremely helpful in corroborating the driving role of the mutated BRAF in melanomagenesis [17]. Moreover, Viros et al. demonstrated in these mice that single or repeated doses of UV radiation, mimicking mild sunburn in humans, induced an accelerated BRAF^V600E^-driven melanomagenesis, strongly supporting the role of UV in melanoma development [18]. However, because of the long latency required for the development of tumors in this GEMM, several groups demonstrated independently that additional genetic lesions could cooperate with the BRAF^V600E^ mutation in accelerating melanoma initiation and progression. Indeed, the association of p16INK4a or p16INK4A/p19ARF loss, as well as, Pten silencing, significantly increase the incidence, reduce the latency and dramatically increase the metastatic behavior of tumors in BRAF^V600E^ GEMM [19,20,21]. More recently, Perna and coauthors generated a GEMM with a conditional expression of BRAF^V618E^ mutation (analogous to BRAF^V600E^ in humans) in mouse melanocytes, supporting the previous findings that oncogenic BRAF alone is sufficient to initiate melanomagenesis, even if with incomplete penetrance and prolonged latency [22]. Notably, the treatment of these mice with a BRAF inhibitor (PLX4720) resulted in tumor regression followed by relapse, mimicking the human clinical condition. And the analysis of recurrent melanomas allowed the identification of novel candidates responsible for the resistance to the BRAF inhibitor PLX4720, with strong potential implications into the human clinics [22]. Dankort et al. took advantages of their BRAF^V600E^; Pten^−/−^ metastatic melanoma GEMM to demonstrate the importance of targeting key signaling pathways linked to these genetic aberrations. Indeed, in this GEMM, the inhibition of mTorc1 by using Rapamycin or of MEK1/2 by using PD325901, prevented melanoma development, but only during drug administration. The results obtained suggested that melanoma-initiating cells survive the treatments and induce tumor relapse as soon as the drug is stopped. Nevertheless, the combined treatment with Rapamycin and PD325901 led to shrinkage of BRAF^V600E^; Pten^−/−^ established melanomas [21].

Overall, these GEMMs provide an excellent system not only to study the role of BRAF^V600E^ in melanoma progression but also to pre-clinically evaluate the consequences of its direct or indirect targeting.

Despite the availability of GEMMs, however, the vast majority of the pre-clinical studies on BRAF^V600E^ role and targeting have been performed in xenograft models, based on the engraftment of human melanoma cells into immunodeficient mice. Taking advantage of these models, early after the identification of the BRAF^V600E^ mutation in human melanomas, the validation of its oncogenic role in the early stage of tumor progression was achieved by expressing BRAF^V600E^ in cultured melanocytes. The expression of the mutated BRAF^V600E^ made the melanocytes capable to give rise to tumors when injected subcutaneously in nude mice, conversely to the BRAF^wt^ counterpart cell line that did not grow [23]. Since then, tons of studies have exploited the xenotransplantation of BRAF^V600E^ human melanoma cells into nude mice which allowed a relatively easy and quick evaluation of the oncogenic role of activated BRAF and the potential of its targeting. Among the most clinically relevant studies, Tsai and collaborators proved the anti-tumor potential of the mutated-BRAF selective inhibitor (PLX4720) in BRAF^V600E^-dependent tumor xenograft models, inducing a significant tumor growth delay and even regression [24]. Later on, several groups, working on different BRAF^V600E^-expressing melanoma xenograft models, reported the striking efficacy of another BRAF inhibitor, PLX4032 (now Vemurafenib), in inducing a partial or complete tumor regression and prolonged animal survival, in a dose-dependent manner and without signs of toxicity [25,26,27]. The positive results obtained by these studies laid the foundation for the Food and Drug Administration (FDA)-approval of Vemurafenib for the treatment of BRAF^V600E^ mutated metastatic melanoma patients, which resulted effective in significantly prolonging the progression-free survival compared with standard therapies [28].

An additional example of a successful translational study from mice to humans is the one demonstrating the anti-tumor activity of another BRAF inhibitor, the Dabrafenib [29], against human melanoma xenografts. These data laid the foundation for Dabrafenib clinical evaluation that finally led to its FDA approval in 2013 for the treatment of metastatic BRAF-mutated melanoma patients [30,31].

### 2.2. Pre-Clinical Modeling of NRAS in Mice

While activating mutations in the BRAF gene were discovered in around 50% of melanoma patients, mutations in NRAS account for 21–28% of melanomas. The vast majority of these mutations (around 84%) are localized in codon 61 (glutamine to arginine; NRAS^Q61R^), while only a small fraction (around 7%) is contained in codon 12 (glycine to aspartic acid; NRAS^G12D^) [32]. As for the BRAF^V600E^, also the importance of NRAS mutations in melanoma development has been studied in GEMMs. In 2005, Ackerman and collaborators demonstrated in vivo that the expression of the constitutively active NRAS^Q61R^ in mouse-melanocytes under the tyrosinase (Tyr) promoter induced cutaneous melanomas in mice, even if with low penetrance and long latency. However, the crossing of these mice into Ink4a/Arf-null background demonstrated the importance of the cooperation of these mutations, since Tyr-NRAS^Q61K^, Ink4a/Arf mice developed metastatic melanomas at higher penetrance (83%) and shorter latency, with lung and liver metastasis [33]. Next, the contribution of activated NRAS oncogene in melanomagenesis, alone or in combination with p16Ink4 or liver kinase B1 (Lkb1) or CDKN2A depletion, was corroborated by other groups [34,35,36]. 

Again, xenograft models were then used to test the efficacy of targeted therapies in NRAS mutated melanomas. However, the direct targeting of NRAS with small-molecule inhibitors is a quite complex approach that has been mostly ineffective, even if the search of a clinically applicable direct RAS-targeted therapy is still active nowadays [37,38]. Since RAS activates the MAPK signaling pathway, the idea to overcome the direct targeting of NRAS by hitting the RAS-MAPK downstream effectors was pre-clinically tested in xenograft models of NRAS mutated melanoma. These experiments laid the foundation for its application in human clinical trials [39,40,41,42].

### 2.3. Pre-Clinical Modeling of NF1 in Mice

The tumor suppressor gene NF1, coding for the neurofibromin protein, has recently emerged as one of the key drivers of melanoma, showing a high-frequency of mutations (12–18% of all melanoma cases) [43,44]. In particular, inactivating NF1 mutations are present in 46% of melanomas expressing wild-type BRAF and RAS [44] and are more commonly observed in chronically sun-exposed skin, desmoplastic melanomas [45,46]. The GTPase-activating domain of NF1 acts as a direct negative regulator of RAS by converting RAS-GTP in RAS-GDP, thus inhibiting downstream RAS signaling. Some missense mutations in NF1 can abolish its normal RAS-GTPase activity with a consequent deregulation of the RAS/MAPK pathway, leading to melanomagenesis. 

Thanks to the generation of a unique GEMM carrying a conditional inactivating mutation in NF1 gene and a conditional activating BRAF^V600E^ mutation (Tyr::CreERT2; Braf^CA/+^; Nf1^flox/flox^ mice), Maertens and colleagues reported that NF1 loss cooperates with BRAF mutation to drive melanomagenesis. Indeed, they described a significant increase in melanoma development rate in Tyr::CreERT2; Braf^CA/+^; Nf1^flox/flox^ mice compared to the BRAF-mutant counterpart [47]. Moreover, NF1 loss in presence of mutant BRAF leads to an ever-increasing hyperpigmentation of the skin and massive melanin deposition with dermis expansion in these mice because of melanocyte hyperproliferation [47]. These data are consistent with previous findings on a transgenic mouse model of NF1 haploinsufficiency, in which melanocytes show higher MAPK and melanin synthesis gene activities than their wild-type counterpart [48].

Thus, NF1 can be qualified as a potent tumor suppressor in melanomas. Furthermore, BRAF/NF1-mutant tumors have turned out to be insensitive to BRAF inhibitors such as Vemurafenib, compared to single BRAF-mutant tumors, both in in vitro and in vivo experiments. This strong implication of NF1 in the drug resistance acquisition is reinforced by the evidence that NF1 mutations are coupled with drug resistance also in a human setting, in BRAF-mutant melanoma patients [43,49,50]. These data suggest the importance of a patients’ selection for an effective treatment. 

### 2.4. The Use of Patient-Derived Xenografts for Targeted Therapies

Almost every patient develops a resistance to targeted therapies. The possibility to gain an insight into the mechanisms of resistance development and possible overcoming is of paramount importance. As a step in this direction, mouse models based on patient-derived tumor xenograft (PDX) have been established [51,52,53]. The idea is to engraft the fresh primary melanoma derived from a patient who is going to be treated directly into immunodeficient mice and to perform a “co-clinical” trial, treating mice ahead or concurrently with the patient. The results obtained from the PDX trial could help in selecting the most accurate treatment for each melanoma patient.

Of course, to be relevant to study the predicted clinical response to therapies, these PDXs should faithfully represent the features of the original patient‘s tumor. For this purpose, melanoma PDXs have been extensively characterized at genomic and clinical-pathological levels.

Several studies revealed a similar genetic pattern between the patients’ original tumors and successive “xenotransplantations” in mice [52,54]. Moreover, Quintana and collaborators demonstrated a comparable metastatic behavior of PDXs derived from patients with stage IIIB/IIIC melanoma, showing that 95% of the generated PDX exhibited metastases to distant organs analogous to those found in the corresponding patient [55]. Interestingly, several reports showed that the use of PDX faithfully reproduces patients’ response to targeted therapies. Thanks to the possibility of generating dozens of mice sampling serial biopsies from a single melanoma patient, a better reproduction of the heterogeneity of the tumor and of the multiple clonally derived mechanisms of resistance to targeted-therapies could be achieved in PDXs [56]. Therefore, PDXs with acquired resistance to BRAF inhibitor can be exploited to pre-clinically identify effective double and triple combination therapies, rationally guiding the use of second-line therapies in patients on the basis of the clinical activity observed in the “personalized” xenograft [56,57,58,59].

Obviously, the possibility to identify the most effective personalized medicine by performing “clinical trial” in PDX models have provoked a great enthusiasm. Nevertheless, this process has some important limitations. One is a matter of time. Indeed, generation of PDXs from a patient’s tumor is not always successful, and when the xenograft develops, it can take several weeks to months to generate sufficient numbers of mouse “avatars”, a time frame that many patients do not have [57,59]. Secondly, since PDXs are generated by implanting human melanomas in immunocompromised mice, they are not good pre-clinical models to evaluate the now well-known important role of the interaction between the tumor and the immune system [60] and to study the efficacy of novel immunotherapeutic approaches [61,62]. Lastly, in 2017, Ben-David and colleagues, by performing a comprehensive genomic analysis evolution in a collection of more than 1000 PDXs of different cancer types, questioned the reliability of PDXs for modeling human therapeutic response [63]. Indeed, their results showed that copy number alterations (CNA) in PDXs and parental lesions are substantially different and that the two hosts follow a distinct selection pressure and a different clonal evolution [63]. 

### 2.5. The Use of the B16 Model to Elucidate the Role of Circulating Exosomes in Metastatic Melanoma

The findings of the presence of extracellular vesicles, in particular of EXs, capable to act as vehicles for the transfer of bioactive molecules between cells, have revolutionized the knowledge of the intercellular communication field [64,65]. Recent advances in cancer research have highlighted interest in the function of EXs as possible players for tumor onset and progression. Indeed, in the oncology field, EXs are known to perform a wide range of fundamental biological and functional roles that influence tumor growth, invasion, metastatization, angiogenesis and stem-cell renewal [66,67,68]. Several studies have shown that melanoma patients have an increased number of EXs in their plasma which could play a role in tumorigenesis and in malignant progression [69]. Particularly, malignant melanoma is characterized by a metastatization process that involves lymphatic dissemination of tumor cells inducing reactive lymphangiogenesis prior to metastatization and eventually promoting the establishment of the pre-metastatic niche within downstream lymph nodes [70,71]. Consequently, melanoma cell-derived EXs could play a critical role at this level, being involved in the alteration of the angiogenic microenvironment of the tumor, eventually resulting in melanoma cells diffusion.

In this regard, the use of the B16 transplantable cell line has allowed elucidating the role of melanoma EXs in promoting disease progression. This widely used cell line was derived from a spontaneous melanoma arose at the base of the ear in a C57BL/6J mouse. B16 transplantable tumors let the study of tumor growth and/or metastatization in immunocompetent syngeneic mice, preserving the interactions between cancer cells and the microenvironment. Several clones with different proliferative and metastasizing capability have been derived from the original cell line [72]; among them, the B16-F10 clone is greatly malignant, with a high metastatic potential to visceral organs, particularly to the lungs. Taking advantage of the in vivo study of EXs derived from B16-F10 cells injected in mice, Peinado and collaborators demonstrated that melanoma EXs could educate bone marrow progenitor cells toward a pro-metastatic phenotype through MET signaling. This observation obtained from pre-clinical studies was then transferred into the human clinical setting, validating the higher level of MET and phospho-MET in circulating EXs isolated from stage III and IV melanoma patients as compared to controls. The results observed suggest that MET expression in circulating EXs could be considered as a new predictor or early marker of metastatic disease in a human clinical setting [73].

## 3. The Weakness of Mouse Models: Testing Immunotherapy against Melanoma

Thanks to the recent advances in deep sequencing, melanomas are now classified at the molecular level depending on the mutational status of their major oncogenic drivers (BRAF, NRAS, and NF1), and are considered as “triple-negative” when no mutations are present in these genes [74]. Accordingly, several targeted inhibitors have been developed and exploited for human melanoma patients’ management. However, after an initial effective response, almost inevitably patients acquire resistance to these targeted therapies through multiple mechanisms [75,76,77] and this still remains a major problem. Novel strategies are therefore urgently needed to overcome resistance to therapies. 

The inherent immunogenicity and the potential role played by immunological events in melanoma natural history have motivated researchers to appraise immunotherapeutic approaches in order to potentiate patients’ own immune system to fight against melanoma cells. When the word “immunotherapy” comes out, the straight thought goes to checkpoint inhibitors (CIs), i.e., monoclonal antibodies directed against the Cytotoxic T Lymphocyte Antigen (CTLA-4) and the Programmed Cell Death Receptor-1 (PD-1) or its ligands. CIs release the brake of the immune system, potentiating patients’ immune cells to fight against the tumor and resulting in impressive clinical benefits. The introduction of the anti-CTLA-4 Ipilimumab in 2011 [78,79], followed by the two anti-PD-1 inhibitors, Nivolumab and Pembrolizumab in 2015 [80,81,82] for melanoma patients’ management has been revolutionary in the immune-oncology field. Clinical trials based on the combination of Nivolumab and Ipilimumab have been recently started, showing better results for the combinatorial approach as compared to CI monotherapy [83,84]. Indeed, with the combination of Nivolumab and Ipilimumab, an overall response rate of 61% was achieved as respect to 11% for Ipilimumab alone, with a progression-free survival of 11.5 months for the combinatorial approach, versus 6.9 and 2.9 months for Nivolumab and Ipilimumab alone, respectively [84]. However, most patients exhibit innate resistance to CIs and suffer from disease progression or severe toxicity. Therefore, improvement and new therapies are needed to “raise the tail” on the survival curve of patients and cancer vaccines may “fuel the engine” and prepare the patient to better respond to CIs.

For the pre-clinical testing of novel cancer immunotherapies, animal models of melanoma have always been exploited. However, if mouse models have undoubtedly contributed to our understanding of molecular mechanisms of melanoma carcinogenesis and to the importance of molecular targeted-therapies, their value in predicting and translating the effectiveness of immunotherapeutic strategies in clinical trials remains controversial. Indeed, over the last years, despite the existence of several successful immunotherapeutic strategies in immunocompetent mouse models, their translation to human clinical oncology failed because of unacceptable toxicity or a lack of efficacy [9,85,86]. Currently, no melanoma vaccine has been approved as a standard treatment for melanoma patients. Possible reasons for the failure to provide benefits could include poor vaccine antigen immunogenicity, ineffective way of vaccine administration and wrong choice of the pre-clinical model. Indeed, despite the unquestionable importance of murine models in human cancer research, the 95% similarity of mouse and human genes cannot compensate for significant species differences in physiology, anatomy, metabolism, biochemistry, pharmacokinetics, and toxicokinetics [87]. Moreover, individual genetic sensitivities, immunologically mediated phenomena, and idiosyncratic reactions in human patients are difficult to reproduce in mouse models. These aspects could be altogether responsible for the inefficient translation of good pre-clinical immunotherapeutic results in new effective immunological therapies for patients. Pro and cons of pre-clinical mouse models of melanoma have been summarized in Table 1.

### Pre-Clinical Testing of Immunotherapies in Mice

Melanoma was the first model to reveal CD4+ and CD8+ cellular specificity to cancer differentiation antigens gp100 and tyrosinase [88,89], suggesting the immunogenicity of the tumor and the ability of the patient’s own immune system to recognize and activate a specific immune response against cancer cells. A great deal of effort has been then put into identifying specific melanoma associated antigens (MAA) to trigger an effective anti-cancer immune response. In this scenario, other melanocyte differentiation antigens, such as MART-1, and cancer-testis antigens, such as MAGE and NY-ESO, were identified [90,91,92,93]. Since then, tons of pre-clinical studies have been performed for the evaluation of the anti-tumor potential of different approaches (i.e., dendritic cells vaccines, peptide vaccines, DNA vaccines and so on) against these well-known MAAs. Almost the totality of these pre-clinical studies has been performed immunizing mice and injecting subcutaneously or intravenously, in a preventive or curative setting, the B16 or B16-F10 melanoma cell lines. Thanks to successful studies performed in this pre-clinical model, several cancer vaccines have been tested, alone or in combination with CIs, in clinical trials [78,94,95,96].

Starting from the observation that murine dendritic cells (DC)-derived EXs were able to induce an antigen-specific CD4+ and CD8+ response in vitro, some pre-clinical studies have been carried out in the B16 mouse model using DC-derived EXs-based vaccination strategies. These EXs are characterized by the expression of several immunologically relevant components, such as MAA-derived peptides in association to MHC class I and II molecules, and co-stimulatory molecules [97,98]. When used to vaccinate mice, a significant protective anti-cancer immunity was induced against a B16 melanoma tumor [99,100]. Escudier et al. in 2005 turned these pre-clinical approaches in a clinical study realizing the first-in-human Phase I trial demonstrating the feasibility of using DC-derived EXs pulsed with MAA-derived peptides for the immunization of stage III/IV melanoma patients [101].

However, in human melanoma patients, the effectiveness of cancer vaccines developed in the B16 mouse model, is only modest, reflecting the difficulty of translating results from mice to humans. Indeed, it can be speculated that the tumors induced by B16 cells transplanted in syngeneic mice do not completely reproduce the cancer-immune system mutual interactions that in humans take place over longer periods as compared to what happen in the transplantable model. This limitation strongly pushes the scientists towards the search for more sophisticated and more valuable translational pre-clinical models and hence the concept of comparative oncology became apparent.

## 4. The Dog Revolution: Canine Tumors as Pre-Clinical Models for Translational Immunotherapy

It was in 2003 that the National Cancer Institute’s Center for Cancer Research promoted the Comparative Oncology Program to foster the use of naturally occurring cancers in pet animals, dogs in particular, as models of human cancer [102]. Soon after a European initiative with a similar purpose—the LUPA project—was launched [103]. Since then, several investigations have been performed to compare the naturally occurring tumors in dogs with the human counterparts [7,104,105]. 

One of the most obvious advantages of canine versus murine models is that dogs spontaneously develop tumors with the same anatomic and physiologic characteristics of human neoplasms, growing over long periods of time in presence of an intact immune system that could deeply interact with cancer cells, shaping the tumor development and progression as occurs in humans. Moreover, pets and owners share the same environment and are exposed to the same carcinogens; a fundamental condition which drives tumor development. The natural evolution of canine tumors follows the human one, with the development of recurrences and metastasis to the same relevant sites, mimicking better than any other pre-clinical model the step-wise progression of human tumors. Canine cancers are more comparable with human neoplasms in terms of size and cell kinetics. Moreover, the inclusion of canine patients from different breeds in clinical trials provides a background of genetic diversity similar to what is seen in human populations.

At the same time, even if there is still a lack of comprehensive investigations, some attempts to compare the anatomical and functional development of the immune system in humans and dogs have been made [106,107,108,109,110]. Interestingly, even if not completely mature to react against the external stress, the immune system of dogs is fully developed before birth, just like happens in humans. Moreover, living in the same environments, and consequently being exposed to the same pathogens and commensals, dogs shares with their owners the typical regulatory T-cell responses which accompany the maturation of the immune system in response to postnatal stimulation. Additionally, the major immune subsets populations, including CD4+, CD8+, CD90+ cells and DC have been characterized in dogs and a significant homology with humans has been demonstrated [111,112]. Conversely, dog NK cells have proved more difficult to be characterized [113]. Interestingly, as largely demonstrated for human patients, the phenotype of lymphocytes in the peripheral blood and tumor microenvironment of dogs with cancer have been linked with prognosis [114,115]. As well, a consistent homology between canine and human immunoglobulins have been demonstrated [116]. All these data reinforce the important homology between dog and human immunobiology, especially in cancer. Of note regarding the immunotherapy studies, the expression of checkpoint molecules, including CTLA-4 and PD-1, has been observed on peripheral blood lymphocytes of several canine patients affected by mastocytoma, melanoma, and renal cell carcinoma. Studies using canine tumor biopsy samples and a human monoclonal antibody that cross-reacts with canine PD-L1 confirm expression of PD-L1 on a number of canine tumors [117,118]. On this basis, studies to explore immune checkpoint blockade in dogs are now under development and a recent clinical trial with a canine-chimeric monoclonal antibody against canine PD-L1 showed a response, even if limited, in dogs with advanced melanoma [119]. All these results make dogs a priceless model for translational immunotherapeutic studies, strongly suggesting that the evaluation of the efficacy of novel immunotherapies in canine patients may be strongly predictive of their clinical efficacy in a human oncological setting (Table 1) [7,8,107].

In the last decades we are witnessing a real “dog revolution” with a far-reaching change in the immune-oncology field: there is an increasing awareness regarding the problem of cancer in canine patients that is becoming a serious challenge as in humans and more and more owners are now demanding advanced care options for their affected dogs [102]. This could represent a turning point for the development of innovative immunotherapies that could benefit canine patients and that at the same time could be rapidly translated to the human clinical setting. 

Among the canine tumors “under the microscope” of the comparative oncology, melanoma is one of the most noteworthy.

### 4.1. Canine Melanoma

Canine melanoma is of strong interest in the comparative oncology because it is a spontaneous, highly aggressive tumor that develops over a long period of time in an immunocompetent microenvironment, as in human patients. Canine melanoma is a relatively common tumor in dogs, with a higher incidence in Cocker Spaniel, Poodle, Pekinese, Gordon Setter, Chow Chow, Golden Retriever, mixed-breed dogs, and Dachshund compared to Boxers and German Shepherds where the melanoma frequency is lower [120]. This suggests that a genetic component is involved in canine melanoma development. Melanoma in dogs can affect different anatomical sites as lips, oral cavity, skin and digit/footpad, being oral melanoma the most aggressive type [105]. Accumulating evidences have highlighted the strong similarities between canine and human melanomas: overlapping cytological, histopathological and architectural features of the tumors have been described for both species [7,105,120,121]. Moreover, several molecular abnormalities and signaling pathways described in specific human melanoma subtypes can be found in canine melanoma, including phosphorylated forms of AKT and ERK1/2, alterations of the receptor tyrosinase KIT and PTEN [104,122,123]. It is noteworthy that the BRAF^V600E^ mutation has not been detected in canine melanomas, suggesting that melanoma-bearing dogs are an ideal model for non-cutaneous or UV-independent melanomagenesis [124]. Even though in humans, melanomas are prevalently cutaneous, they could arise from tissues other than the skin, including the uvea of the eye (5.2%) and within mucous membranes (1.3%), with a similar incidence independently from the phototypes and therefore with a higher incidence in many part of the world [125,126]. And also, among human cutaneous melanomas, there is a subgroup characterized by the lack of the most common recurring mutations of BRAF, N/H/K-RAS, or NF1, which are referred to as “triple negative” or “triple wild-type” subtype [74]. This triple negative molecular genotype is also a feature of mucosal melanomas, which behave more aggressively and have the worst prognosis as compared to other melanoma subtypes [127]. Therefore, for their characteristics, canine oral melanomas have been proposed as invaluable resources for in vivo investigation of mucosal and triple-negative melanoma [128].

Canine oral melanoma accounts for 30–40% of all oral tumors, estimating up to 100,000 diagnoses each year in the USA [104,105]. It is the most exploited for comparative oncology studies due to its highly metastatic attitude, with 80% of dogs presenting tumor invasion in different organs, including regional lymph nodes and lungs, which are relevant sites for melanoma metastatization in human patients [104,105]. Similarly to the poor prognosis of human malignant melanoma patients, the median survival time of dogs affected by oral melanomas is very short, being of 200 days after diagnosis, with a high mortality rate because of recurrences and metastasis [7,129]. Standard therapies are the same as in human patients i.e., mostly surgery, radiotherapy and/or chemotherapy. For canine patients, surgery and radiotherapy are the main treatment options for local control of the tumor, which occurs in about 75% of treated dog. However, the 1-year overall survival is less than 30% due to metastasis development [129]. For this reason, systemic chemotherapy could be considered as a valid option even if it has very little, if none, benefits in terms of clinical outcome as compared to the local control of the disease [130]. Therefore, from the clinical point of view, innovative and more effective treatments are still needed for both canine and human melanoma, because of their chemo- and radio-resistance. On one hand, already approved human therapies (i.e., CIs) could be used to treat also canine melanoma, while on the other, new therapies developed in this excellent translational melanoma model could be speedily translated for the management of human patients. 

### 4.2. Immunotherapy in Canine Melanoma

Following the ambition to develop new effective treatments, several groups performed veterinary clinical trials evaluating the anti-tumor potential of different immunotherapeutic strategies mainly focused on cytokine-based or antigen-based approaches. 

The anti-tumor potential of several cytokines such as interleukin 2 (IL-2), interferon γ (IFN-γ), granulocyte-macrophage colony-stimulating factor (GM-CSF), IL-12 and IL-18 have been evaluated for the treatment of canine melanoma patients. Different approaches have been used to evaluate the effectiveness of these cytokines-based therapies, ranging from administration of genetically engineered xenogeneic or autologous cytokines-producing tumor cells, plasmid lipofection or naked plasmids administration. In general, all these studies demonstrated the potential of these immunotherapeutic approaches in prolonging survival of canine melanoma patients when applied in an adjuvant setting.

One of the pioneering work was published more than 20 years ago by Quintin-Colonna and collaborators, describing the efficacy of repeated local injections of human IL-2-genetically engineered xenogeneic cells at the primary tumor site after its surgical removal in association to radiotherapy [131]. A few years later, Hogge and coworkers demonstrated the potential of the intradermal delivery of irradiated autologous tumor cells engineered to produce the human GM-CSF in canine melanoma patients after the surgical resection of the tumor [132]. Since then, several additional veterinary trials have been performed evaluating the potential of these two cytokines alone or in combination with “suicide genes” such as the herpes simplex virus thymidine kinase (HSV-TK) or with IFN-γ [133,134].

Since the 1980s, the anti-tumor potential of the same cytokines described above have been investigated in large-scale clinical trials for the treatment of human melanoma patients [135,136,137], leading to the FDA approval of IL-2 for cancer treatment of melanomas that are generally refractory to standard therapy, and to the extensive testing of other cytokines, including GM-CSF, IL-12, and IL-18 for patients with advanced cancer. 

For both humans and dogs, even if it is noteworthy that some patients with disseminated disease achieved durable clinical benefits, the toxicities of these approaches suggested the need of identifying a better therapeutic schedule for the treatment of both species. 

A different immunotherapeutic strategy could be that focused on antigen-based therapies, mostly using DNA vaccines against MAA shared between canine and human melanoma patients. The most common MAA investigated in veterinary clinical trials are the tyrosinase and gp100 antigens, alone or in combination with cytokines [138,139,140,141,142]. 

A first Phase I pilot veterinary trial exploiting the safety and the anti-tumor potential of a xenogeneic DNA vaccine coding for the human tyrosinase in dogs affected by advanced malignant melanoma was conducted in 2003 by Bergman and collaborators [138]. This small study suggested for the first time that the intramuscular anti-tyrosinase DNA vaccination was safe in dogs and endowed with a clinical potential, laying the foundation for further investigations. Later on, additional veterinary trials demonstrated that this DNA vaccination strategy could indeed have a therapeutic efficacy with better results in stage II/III local-controlled canine melanoma patients [140,143]. These positive results, along with the demonstration of the induction of a specific anti-tyrosinase immune response in some DNA vaccinated dogs [139], led, in 2010, to the approval by the United States Department of Agriculture (USDA) of the first anti-human tyrosinase DNA vaccine (Oncept, Merial) for the treatment of melanoma affected dogs. Moreover, thanks to these veterinary results, a rapid translation of the proposed therapeutic approach to the human clinical setting was achieved. Indeed, soon after, clinical trials in human advanced melanoma patients started as well, exploiting the same strategy of xenogeneic DNA vaccination against the tyrosinase antigen [144,145]. 

However, with the coming out of the most recent results from multiple veterinary trials including a large canine melanoma population, the therapeutic efficacy of Oncept has been questioned [141,146]. The inconclusive results about the therapeutic effectiveness of Oncept in dogs could be related to the feature of the target antigen and to the design of the clinical trials, which included both tyrosinase positive and negative canine patients. In-human trials, the safety and the immunogenicity of the vaccination protocol were proved. However, as well as in canine patients, a significant clinical activity was not reached, confirming a comparable outcome in trials performed in the two species. 

Nevertheless, the approval of Oncept as the first anti-cancer DNA vaccine and the demonstration of the safety and immunogenicity of xenogeneic DNA vaccination both in dogs and humans have spurred several groups and us to investigate the translational efficacy of the immune-targeting of other antigens relevant for human and canine melanoma patients [147,148,149]. As a step in this direction, we focused our attention on one of the most interesting MAA identified so far, the chondroitin sulfate proteoglycan 4 (CSPG4). It is a prototype “oncoantigen” in human melanomas, since it has a restricted expression in healthy/normal tissues, but a high expression on melanoma cells in almost 85% of patients [150,151]. The CSPG4 has a key role in regulating the proliferative, migratory, invasive and metastatizing ability of cancer cells and their chemoresistance, therefore simultaneously regulating several processes needed for cancer development and progression. Moreover, CSPG4 expression has been corroborated not only on differentiated cancer cells but also on cancer stem/initiating cells (CIC), considered responsible for recurrences and metastasis, therefore making the CSPG4 an even more interesting target for immunotherapy. 

On these bases, we identified CSPG4 as a new marker for canine patients affected by spontaneous oral melanoma [151,152]. We performed two veterinary trials to evaluate the potential of a xenogeneic DNA vaccine coding for the human CSPG4 in canine melanoma patients affected by spontaneous stage II-III CSPG4-positive oral melanomas. Dogs were electrovaccinated (DNA vaccination was associated with electroporation) monthly after the surgical removal of the primary tumor, in order to prevent recurrence and metastasis. This adjuvant vaccination was effective in breaking the immune tolerance against the self-antigen, resulting in the induction of an immune response against the canine CSPG4 and in the significant prolongation of the survival of vaccinated patients as compared to controls treated with conventional therapies alone [148,149]. 

Finally, given the importance of spontaneous canine cancers, recently circulating EXs have been evaluated also in dogs. Canine EXs are characterized by a size range and a protein cargo similar to human EXs [153,154]. Literature, unfortunately, provides a restricted set of information on the investigation of canine EXs, opening up a great chance for new and interesting studies on EXs in canine melanoma patients for translational applications.

## 5. Conclusions

**Figure 1:**
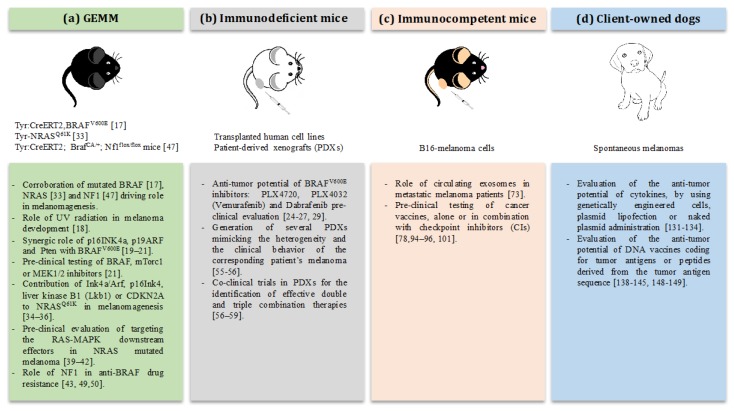
Most relevant achievements in the melanoma onco-immunology field obtained in (**a**) genetically engineered mouse models (GEMM); (**b**) immunodeficient mice xenotransplanted with human cell lines or patient-derived tumors (PDXs); (**c**) immunocompetent mice challenged with syngeneic B16 cells; (**d**) canine patients affected by spontaneous melanoma.

In conclusion, several pre-clinical models for the modelling of melanoma have been developed. The mouse models are good examples to elucidate the relevance of specific driver genes for melanomagenesis, further providing an indispensable platform for the pre-clinical testing of drug efficacy and resistance mechanisms (Figure 1).

Nonetheless, in the tumor immunology field, given the complexity of melanoma and immune system interactions, pre-clinical mouse models of cancers have been considered poor prognostic, being not predictive of the results in the human clinical setting, as shown by the high rate failure of their translation in the human clinics. 

It is nowadays widely recognized that spontaneous cancers in dogs represent a highly translational model. They offer the opportunity to investigate the clinical potential of novel immunotherapies in a comprehensive way (Figure 1), including the “scaling up” of doses, taking into account the presence of a complex and long-lasting interaction between the tumors and immune cells, and the possibility of long-term assessment of efficacy and toxicity. All these features may allow the building of solid foundations for the rapid translation of the results from melanoma canine patients to human melanoma patients’ management, with a benefit for both species.

## Figures and Tables

**Table 1:** Strengths and weaknesses of the translational value of melanoma pre-clinical models.

	Model	Strengths	Weaknesses	References
**In vitro**	**Cultured cell lines**	✓ Easy to handle ✓ Major reproducibility	✓ Lack of three-dimensional architecture ✓ Absence of interaction with the microenvironment and with the immune system ✓ No heterogeneity	*Brandner* *et al., 2013* [4] *Turley* *et al., 2015* [5]
**In vivo**	**Genetically Engineered Mouse Model: BRAF^V600E^; NRAS; NF1**	✓ Specific gene mutation ✓ Combination of multiple gene mutations ✓ Functional immune system ✓ Stepwise tumor progression	✓ Long latency ✓ Incomplete penetrance ✓ Different anatomy, physiology and biochemistry compared to human ✓ Lack of different genetic background	*Perna* *et al., 2015* [22] *Talmadge et al., 2007* [87]
	**Transplantable tumor model**	✓ Functional immune system ✓ Tumor interaction with the microenvironment ✓ Metastasis formation	✓ Less predictive for the clinical translation ✓ Different anatomy, physiology and biochemistry compared to human ✓ Not properly reproducing the interactions between cancer cells and the immune system	*Riccardo et al., 2014* [7] *Talmadge et al., 2007* [87]
	**Patient-derived xenograft in immunodeficient mouse models**	✓ Use of human tumor samples ✓ Heterogeneity ✓ Metastasis formation ✓ Possibilities for “co-clinical trials” ✓ Study of drug resistance	✓ Absence of interactions with the immune system ✓ Long latency for tumor growth ✓ Different tumor evolution as compared to parental lesion	*Hidalgo* *et al., 2011* [57] *Krepler et al., 2016* [59] *Hylander et al., 2013* [60] *Pickup et al., 2014* [61] *Hartsough et al., 2016* [62] *Ben-David et al., 2017* [63]
	**(Client owned)-Dogs**	✓ Shared environment with humans ✓ Spontaneous tumor formation ✓ Functional immune system ✓ Recurrence and metastasis ✓ Different genetic background	✓ Poor knowledge and understanding of the immune system	*Riccardo et al., 2014* [7] *Riccardo et al., 2016* [8]

## Abbreviations

BAP1	BRCA1 associated protein 1
CIC	cancer stem/initiating cells
CIs	checkpoint inhibitors
CNA	copy number alterations
CSPG4	chondroitin sulfate proteoglycan 4
CTLA-4	Cytotoxic T Lymphocyte Antigen
DC	dendritic cells
EXs	exosomes
FDA	Food and Drug Administration
GEM	genetically engineered mouse model
GM-CSF	granulocyte-macrophage colony-stimulating factor
GNA11	guanine-nucleotide binding protein G subunit alpha 11
GNAQ	guanine-nucleotide binding protein G subunit alpha Q
HSV-TK	herpes simplex virus thymidine kinase
IFN-γ	interferon γ
IL-2	interleukin 2
Lkb1	liver kinase B1
MAA	melanoma associated antigens
PD-1	Programmed Cell Death Receptor-1
PDX	patient-derived tumor xenograft
SF3B1	splicing factor 3b subunit 1
Tyr	tyrosinase
TRP2	tyrosinase related protein 2
USDA	United States Department of Agriculture

## References

[1] Siegel R.L., Miller K.D., Jemal A. (2017). Cancer statistics, 2017. CA Cancer J. Clin..

[2] Sandru A., Voinea S., Panaitescu E., Blidaru A. (2014). Survival rates of patients with malignant melanoma. J. Med. Life.

[3] Shaikh W.R., Dusza S.W., Weinstock M.A., Oliveria S.A., Geller A.C., Halpern A.C. (2016). Melanoma Thickness and Survival Trends in the United States, 1989–2009. J. Natl. Cancer Inst..

[4] Brandner J.M., Haass N.K. (2013). Melanoma’s connections to the tumour microenvironment. Pathology.

[5] Turley S.J., Cremasco V., Astarita J.L. (2015). Immunological hallmarks of stromal cells in the tumour microenvironment. Nat. Rev. Immunol..

[6] Beaumont K., Mohana-Kumaran N., Haass N. (2013). Modeling Melanoma In Vitro and In Vivo. Healthcare.

[7] Riccardo F., Aurisicchio L., Impellizeri J.A., Cavallo F. (2014). The importance of comparative oncology in translational medicine. Cancer Immunol. Immunother..

[8] Riccardo F., Rolih V., Cavallo F. (2016). Animal models for translational cancer immunotherapy studies. IFMBE Proceedings.

[9] Kamb A. (2005). What’s wrong with our cancer models?. Nat. Rev. Drug Discov..

[10] Shain A.H., Yeh I., Kovalyshyn I., Sriharan A., Talevich E., Gagnon A., Dummer R., North J., Pincus L., Ruben B. (2015). The Genetic Evolution of Melanoma from Precursor Lesions. N. Engl. J. Med..

[11] McKinney A.J., Holmen S.L. (2011). Animal models of melanoma: A somatic cell gene delivery mouse model allows rapid evaluation of genes implicated in human melanoma. Chin. J. Cancer.

[12] Davies H., Bignell G.R., Cox C., Stephens P., Edkins S., Clegg S., Teague J., Woffendin H., Garnett M.J., Bottomley W. (2002). Mutations of the BRAF gene in human cancer. Nature.

[13] Curtin J.A., Fridlyand J., Kageshita T., Patel H.N., Busam K.J., Kutzner H., Cho K.-H., Aiba S., Bröcker E.-B., LeBoit P.E. (2005). Distinct Sets of Genetic Alterations in Melanoma. N. Engl. J. Med..

[14] Caramel J., Papadogeorgakis E., Hill L., Browne G.J., Richard G., Wierinckx A., Saldanha G., Osborne J., Hutchinson P., Tse G. (2013). A Switch in the Expression of Embryonic EMT-Inducers Drives the Development of Malignant Melanoma. Cancer Cell.

[15] Wellbrock C., Rana S., Paterson H., Pickersgill H., Brummelkamp T., Marais R. (2008). Oncogenic BRAF regulates melanoma proliferation through the lineage specific factor MITF. PLoS ONE.

[16] Pollock P.M., Harper U.L., Hansen K.S., Yudt L.M., Stark M., Robbins C.M., Moses T.Y., Hostetter G., Wagner U., Kakareka J. (2003). High frequency of BRAF mutations in nevi. Nat. Genet..

[17] Dhomen N., Reis-Filho J.S., da Rocha Dias S., Hayward R., Savage K., Delmas V., Larue L., Pritchard C., Marais R. (2009). Oncogenic Braf Induces Melanocyte Senescence and Melanoma in Mice. Cancer Cell.

[18] Viros A., Sanchez-Laorden B., Pedersen M., Furney S.J., Rae J., Hogan K., Ejiama S., Girotti M.R., Cook M., Dhomen N. (2014). Ultraviolet radiation accelerates BRAF-driven melanomagenesis by targeting TP53. Nature.

[19] Goel V.K., Ibrahim N., Jiang G., Singhal M., Fee S., Flotte T., Westmoreland S., Haluska F.S., Hinds P.W., Haluska F.G. (2009). Melanocytic nevus-like hyperplasia and melanoma in transgenic BRAFV600E mice. Oncogene.

[20] Hooijkaas A.I., Gadiot J., van der Valk M., Mooi W.J., Blank C.U. (2012). Targeting BRAFV600E in an inducible murine model of melanoma. Am. J. Pathol..

[21] Dankort D., Curley D.P., Cartlidge R.A., Nelson B., Karnezis A.N., Damsky W.E., You M.J., DePinho R.A., McMahon M., Bosenberg M. (2009). BrafV600Ecooperates with Pten loss to induce metastatic melanoma. Nat. Genet..

[22] Perna D., Karreth F.A., Rust A.G., Perez-Mancera P.A., Rashid M., Iorio F., Alifrangis C., Arends M.J., Bosenberg M.W., Bollag G. (2015). BRAF inhibitor resistance mediated by the AKT pathway in an oncogenic BRAF mouse melanoma model. Proc. Natl. Acad. Sci. USA.

[23] Wellbrock C., Ogilvie L., Hedley D., Karasarides M., Martin J., Niculescu-Duvaz D., Springer C.J., Marais R. (2004). V599EB-RAF is an Oncogene in Melanocytes. Cancer Res..

[24] Tsai J., Lee J.T., Wang W., Zhang J., Cho H., Mamo S., Bremer R., Gillette S., Kong J., Haass N.K. (2008). Discovery of a selective inhibitor of oncogenic B-Raf kinase with potent antimelanoma activity. Proc. Natl. Acad. Sci. USA.

[25] Lee J.T., Li L., Brafford P.A., Van Den Eijnden M., Halloran M.B., Sproesser K., Haass N.K., Smalley K.S.M., Tsai J., Bollag G. (2010). PLX4032, a potent inhibitor of the B-Raf V600E oncogene, selectively inhibits V600E-positive melanomas. Pigment Cell Melanoma Res..

[26] Yang H., Higgins B., Kolinsky K., Packman K., Go Z., Iyer R., Kolis S., Zhao S., Lee R., Grippo J.F. (2010). RG7204 (PLX4032), a selective BRAFV600Einhibitor, displays potent antitumor activity in preclinical melanoma models. Cancer Res..

[27] Bollag G., Hirth P., Tsai J., Zhang J., Ibrahim P.N., Cho H., Spevak W., Zhang C., Zhang Y., Habets G. (2010). Clinical efficacy of a RAF inhibitor needs broad target blockade in BRAF-mutant melanoma. Nature.

[28] Mcarthur G.A., Chapman P.B., Robert C., Larkin J., Haanen J.B., Dummer R., Ribas A., Hogg D., Hamid O., Ascierto P.A. (2014). Safety and efficacy of vemurafenib in BRAF V600E and BRAF V600K mutation-positive melanoma (BRIM-3): Extended follow-up of a phase 3, randomised, open-label study. Lancet Oncol..

[29] Rheault T.R., Stellwagen J.C., Adjabeng G.M., Hornberger K.R., Petrov K.G., Waterson A.G., Dickerson S.H., Mook R.A., Laquerre S.G., King A.J. (2013). Discovery of dabrafenib: A selective inhibitor of Raf Kinases with antitumor activity against B-Raf-driven tumors. ACS Med. Chem. Lett..

[30] King A.J., Arnone M.R., Bleam M.R., Moss K.G., Yang J., Fedorowicz K.E., Smitheman K.N., Erhardt J.A., Hughes-Earle A., Kane-Carson L.S. (2013). Dabrafenib; Preclinical Characterization, Increased Efficacy when Combined with Trametinib, while BRAF/MEK Tool Combination Reduced Skin Lesions. PLoS ONE.

[31] Hauschild A., Grob J.J., Demidov L.V., Jouary T., Gutzmer R., Millward M., Rutkowski P., Blank C.U., Miller W.H., Kaempgen E. (2012). Dabrafenib in BRAF-mutated metastatic melanoma: A multicentre, open-label, phase 3 randomised controlled trial. Lancet.

[32] Zhang T., Dutton-Regester K., Brown K.M., Hayward N.K. (2016). The genomic landscape of cutaneous melanoma. Pigment Cell Melanoma Res..

[33] Ackermann J., Frutschi M., Kaloulis K., McKee T., Trumpp A., Beermann F. (2005). Metastasizing melanoma formation caused by expression of activated N-RasQ61K on an INK4a-deficient background. Cancer Res..

[34] Burd C.E., Liu W., Huynh M.V., Waqas M.A., Gillahan J.E., Clark K.S., Fu K., Martin B.L., Jeck W.R., Souroullas G.P. (2014). Mutation-specific RAS oncogenicity explains NRAS codon 61 selection in melanoma. Cancer Discov..

[35] Liu W., Monahan K.B., Pfefferle A.D., Shimamura T., Sorrentino J., Chan K.T., Roadcap D.W., Ollila D.W., Thomas N.E., Castrillon D.H. (2012). LKB1/STK11 Inactivation Leads to Expansion of a Prometastatic Tumor Subpopulation in Melanoma. Cancer Cell.

[36] Kwong L.N., Costello J.C., Liu H., Jiang S., Helms T.L., Langsdorf A.E., Jakubosky D., Genovese G., Muller F.L., Jeong J.H. (2012). Oncogenic NRAS signaling differentially regulates survival and proliferation in melanoma. Nat. Med..

[37] Marcus K., Mattos C. (2015). Direct attack on RAS: Intramolecular communication and mutation-specific effects. Clin. Cancer Res..

[38] Cox A.D., Der C.J., Philips M.R. (2015). Targeting RAS membrane association: Back to the future for anti-RAS drug discovery?. Clin. Cancer Res..

[39] Boespflug A., Caramel J., Dalle S., Thomas L. (2017). Treatment of NRAS-mutated advanced or metastatic melanoma: Rationale, current trials and evidence to date. Ther. Adv. Med. Oncol..

[40] Posch C., Cholewa B.D., Vujic I., Sanlorenzo M., Ma J., Kim S.T., Kleffel S., Schatton T., Rappersberger K., Gutteridge R. (2015). Combined Inhibition of MEK and Plk1 Has Synergistic Antitumor Activity in NRAS Mutant Melanoma. J. Invest. Dermatol..

[41] Vogel C.J., Smit M.A., Maddalo G., Possik P.A., Sparidans R.W., van der Burg S.H., Verdegaal E.M., Heck A.J.R., Samatar A.A., Beijnen J.H. (2015). Cooperative induction of apoptosis in NRAS mutant melanoma by inhibition of MEK and ROCK. Pigment Cell Melanoma Res..

[42] Ascierto P.A., Schadendorf D., Berking C., Agarwala S.S., van Herpen C.M.L., Queirolo P., Blank C.U., Hauschild A., Beck J.T., St-Pierre A. (2013). MEK162 for patients with advanced melanoma harbouring NRAS or Val600 BRAF mutations: A non-randomised, open-label phase 2 study. Lancet Oncol..

[43] Kiuru M., Busam K.J. (2017). The NF1 gene in tumor syndromes and melanoma. Lab. Investig..

[44] Krauthammer M., Kong Y., Bacchiocchi A., Evans P., Pornputtapong N., Wu C., McCusker J.P., Ma S., Cheng E., Straub R. (2015). Exome sequencing identifies recurrent mutations in NF1 and RASopathy genes in sun-exposed melanomas. Nat. Genet..

[45] Wiesner T., Kiuru M., Scott S.N., Arcila M., Halpern A.C., Hollmann T., Berger M.F., Busam K.J. (2015). NF1 mutations are common in desmoplastic melanoma. Am. J. Surg. Pathol..

[46] Shain A.H., Garrido M., Botton T., Talevich E., Yeh I., Sanborn J.Z., Chung J., Wang N.J., Kakavand H., Mann G.J. (2015). Exome sequencing of desmoplastic melanoma identifies recurrent NFKBIE promoter mutations and diverse activating mutations in the MAPK pathway. Nat. Genet..

[47] Maertens O., Johnson B., Hollstein P., Frederick D.T., Cooper Z.A., Messiaen L., Bronson R.T., McMahon M., Granter S., Flaherty K. (2013). Elucidating distinct roles for NF1 in melanomagenesis. Cancer Discov..

[48] Diwakar G., Zhang D., Jiang S., Hornyak T.J. (2008). Neurofibromin as a regulator of melanocyte development and differentiation. J. Cell Sci..

[49] Jousma E., Rizvi T.A., Wu J., Janhofer D., Dombi E., Dunn R.S., Kim M.O., Masters A.R., Jones D.R., Cripe T.P. (2015). Preclinical assessments of the MEK inhibitor PD-0325901 in a mouse model of Neurofibromatosis type 1. Pediatr. Blood Cancer.

[50] Karajannis M.A., Ferner R.E. (2015). Neurofibromatosis-related tumors: Emerging biology and therapies. Curr. Opin. Pediatr..

[51] Sausville E.A., Burger A.M. (2006). Contributions of human tumor xenografts to anticancer drug development. Cancer Res..

[52] Tentler J.J., Tan A.C., Weekes C.D., Jimeno A., Leong S., Pitts T.M., Arcaroli J.J., Messersmith W.A., Eckhardt S.G. (2012). Patient-derived tumour xenografts as models for oncology drug development. Nat. Rev. Clin. Oncol..

[53] Krepler C., Sproesser K., Brafford P., Beqiri M., Garman B., Xiao M., Shannan B., Watters A., Perego M., Zhang G. (2017). A Comprehensive Patient-Derived Xenograft Collection Representing the Heterogeneity of Melanoma. Cell Rep..

[54] Einarsdottir B.O., Bagge R.O., Bhadury J., Jespersen H., Mattsson J., Nilsson L.M., Truvé K., López M.D., Naredi P., Nilsson O. (2014). Melanoma patient-derived xenografts accurately model the disease and develop fast enough to guide treatment decisions. Oncotarget.

[55] Quintana E., Piskounova E., Shackleton M., Weinberg D., Eskiocak U., Fullen D.R., Johnson T.M., Morrison S.J. (2012). Human melanoma metastasis in NSG mice correlates with clinical outcome in patients. Sci. Transl. Med..

[56] Das Thakur M., Salangsang F., Landman A.S., Sellers W.R., Pryer N.K., Levesque M.P., Dummer R., McMahon M., Stuart D.D. (2013). Modelling vemurafenib resistance in melanoma reveals a strategy to forestall drug resistance. Nature.

[57] Hidalgo M., Bruckheimer E., Rajeshkumar N.V., Garrido-Laguna I., De Oliveira E., Rubio-Viqueira B., Strawn S., Wick M.J., Martell J., Sidransky D. (2011). A pilot clinical study of treatment guided by personalized tumorgrafts in patients with advanced cancer. Mol. Cancer Ther..

[58] Monsma D.J., Cherba D.M., Eugster E.E., Dylewski D.L., Davidson P.T., Peterson C.A., Borgman A.S., Winn M.E., Dykema K.J., Webb C.P. (2015). Melanoma patient derived xenografts acquire distinct vemurafenib resistance mechanisms. Am. J. Cancer Res..

[59] Krepler C., Xiao M., Sproesser K., Brafford P.A., Shannan B., Beqiri M., Liu Q., Xu W., Garman B., Nathanson K.L. (2016). Personalized preclinical trials in BRAF Inhibitor-resistant patient-derived xenograft models identify second-line combination therapies. Clin. Cancer Res..

[60] Hylander B.L., Punt N., Tang H., Hillman J., Vaughan M., Bshara W., Pitoniak R., Repasky E.A. (2013). Origin of the vasculature supporting growth of primary patient tumor xenografts. J. Transl. Med..

[61] Pickup M.W., Mouw J.K., Weaver V.M. (2014). The extracellular matrix modulates the hallmarks of cancer. EMBO Rep..

[62] Hartsough E.J., Aplin A.E. (2016). Of Mice and melanoma: PDX system for modeling personalized medicine. Clin. Cancer Res..

[63] Ben-David U., Ha G., Tseng Y.Y., Greenwald N.F., Oh C., Shih J., McFarland J.M., Wong B., Boehm J.S., Beroukhim R. (2017). Patient-derived xenografts undergo mouse-specific tumor evolution. Nat. Genet..

[64] Tkach M., Théry C. (2016). Communication by Extracellular Vesicles: Where We Are and Where We Need to Go. Cell.

[65] Tkach M., Kowal J., Théry C. (2018). Why the need and how to approach the functional diversity of extracellular vesicles. Philos. Trans. R. Soc. Lond. B Biol. Sci..

[66] D’Souza-Schorey Crislyn C., Clancy J.W. (2012). Tumor-derived microvesicles: Shedding light on novel microenvironment modulators and prospective cancer biomarkers. Genes Dev..

[67] Miller I.V., Grunewald T.G.P. (2015). Tumour-derived exosomes: Tiny envelopes for big stories. Biol. Cell.

[68] Wu J., Qu Z., Fei Z.-W., Wu J.-H., Jiang C.-P. (2017). Role of stem cell-derived exosomes in cancer. Oncol. Lett..

[69] Logozzi M., De Milito A., Lugini L., Borghi M., Calabrò L., Spada M., Perdicchio M., Marino M.L., Federici C., Iessi E. (2009). High levels of exosomes expressing CD63 and caveolin-1 in plasma of melanoma patients. PLoS ONE.

[70] Rinderknecht M., Detmar M. (2008). Tumor lymphangiogenesis and melanoma metastasis. J. Cell. Physiol..

[71] Hood J.L., San Roman S., Wickline S.A. (2011). Exosomes released by melanoma cells prepare sentinel lymph nodes for tumor metastasis. Cancer Res..

[72] Fidler I.J. (1973). Selection of successive tumour lines for metastasis. Nat. New Biol..

[73] Peinado H., Alečković M., Lavotshkin S., Matei I., Costa-Silva B., Moreno-Bueno G., Hergueta-Redondo M., Williams C., García-Santos G., Ghajar C.M. (2012). Melanoma exosomes educate bone marrow progenitor cells toward a pro-metastatic phenotype through MET. Nat. Med..

[74] The Cancer Genome Atlas Network (2015). Genomic Classification of Cutaneous Melanoma. Cell.

[75] Spagnolo F., Ghiorzo P., Queirolo P. (2014). Overcoming resistance to BRAF inhibition in BRAF-mutated metastatic melanoma. Oncotarget.

[76] Brighton H.E., Angus S.P., Bo T., Roques J., Tagliatela A.C., Darr D., Karagoz K., Sciaky N., Gatza M., Sharpless N.E. (2018). New mechanisms of resistance to MEK inhibitors in melanoma revealed by intravital imaging. Cancer Res..

[77] Manzano J.L., Layos L., Bugés C., de los Llanos Gil M., Vila L., Martínez-Balibrea E., Martínez-Cardús A. (2016). Resistant mechanisms to BRAF inhibitors in melanoma. Ann. Transl. Med..

[78] Hodi F.S., O’Day S.J., McDermott D.F., Weber R.W., Sosman J.A., Haanen J.B., Gonzalez R., Robert C., Schadendorf D., Hassel J.C. (2010). Improved survival with ipilimumab in patients with metastatic melanoma. N. Engl. J. Med..

[79] Robert C., Thomas L., Bondarenko I., O’Day S., Weber J., Garbe C., Lebbe C., Baurain J.-F., Testori A., Grob J.-J. (2011). Ipilimumab plus Dacarbazine for Previously Untreated Metastatic Melanoma. N. Engl. J. Med..

[80] Patnaik A., Kang S.P., Rasco D., Papadopoulos K.P., Elassaiss-Schaap J., Beeram M., Drengler R., Chen C., Smith L., Espino G. (2015). Phase I study of pembrolizumab (MK-3475; Anti-PD-1 monoclonal antibody) in patients with advanced solid tumors. Clin. Cancer Res..

[81] Brahmer J.R., Tykodi S.S., Chow L.Q.M., Hwu W.-J., Topalian S.L., Hwu P., Drake C.G., Camacho L.H., Kauh J., Odunsi K. (2012). Safety and Activity of Anti–PD-L1 Antibody in Patients with Advanced Cancer. N. Engl. J. Med..

[82] Topalian S.L., Hodi F.S., Brahmer J.R., Gettinger S.N., Smith D.C., McDermott D.F., Powderly J.D., Carvajal R.D., Sosman J.A., Atkins M.B. (2012). Safety, Activity, and Immune Correlates of Anti–PD-1 Antibody in Cancer. N. Engl. J. Med..

[83] Postow M.A., Chesney J., Pavlick A.C., Robert C., Grossmann K., McDermott D., Linette G.P., Meyer N., Giguere J.K., Agarwala S.S. (2015). Nivolumab and Ipilimumab Versus Ipilimumab in Untreated Melanoma. N. Engl. J. Med..

[84] Larkin J., Chiarion-Sileni V., Gonzalez R., Grob J.J., Cowey C.L., Lao C.D., Schadendorf D., Dummer R., Smylie M., Rutkowski P. (2015). Combined Nivolumab and Ipilimumab or Monotherapy in Untreated Melanoma. N. Engl. J. Med..

[85] Kamb A., Wee S., Lengauer C. (2007). Why is cancer drug discovery so difficult?. Nat. Rev. Drug Discov..

[86] Tan A.C.L., Goubier A., Kohrt H.E. (2015). A quantitative analysis of therapeutic cancer vaccines in phase 2 or phase 3 trial. J. Immunother. Cancer.

[87] Talmadge J.E., Singh R.K., Fidler I.J., Raz A. (2007). Murine Models to Evaluate Novel and Conventional Therapeutic Strategies for Cancer. Am. J. Pathol..

[88] Touloukian C.E., Leitner W.W., Topalian S.L., Li Y.F., Robbins P.F., Rosenberg S.A., Restifo N.P. (2000). Identification of a MHC class II-restricted human gp100 epitope using DR4-IE transgenic mice. J. Immunol..

[89] Topalian S.L., Rivoltini L., Mancini M., Ng J., Hartzman R.J., Rosenberg S.A. (1994). Melanoma-specific CD4+ T lymphocytes recognize human melanoma antigens processed and presented by epstein-barr virus-transformed B cells. Int. J. Cancer.

[90] Kawakami Y., Eliyahu S., Sakaguchi K., Robbins P.F., Rivoltini L., Yannelli J.R., Appella E., Rosenberg S.A. (1994). Identification of the immunodominant peptides of the MART-1 human melanoma antigen recognized by the majority of HLA-A2-restricted tumor infiltrating lymphocytes. J. Exp. Med..

[91] Van der Bruggen P., Traversari C., Chomez P., Lurquin C., De Plaen E., Van den Eynde B., Knuth A., Boon T. (1991). A gene encoding an antigen recognized by cytolytic T lymphocytes on a human melanoma. Science.

[92] Sang M., Wang L., Ding C., Zhou X., Wang B., Wang L., Lian Y., Shan B. (2011). Melanoma-associated antigen genes—An update. Cancer Lett..

[93] Schultz-Thater E., Noppen C., Gudat F., Dürmüller U., Zajac P., Kocher T., Heberer M., Spagnoli G.C. (2000). NY-ESO-1 tumour associated antigen is a cytoplasmic protein detectable by specific monoclonal antibodies in cell lines and clinical specimens. Br. J. Cancer.

[94] Schwartzentruber D.J., Lawson D.H., Richards J.M., Conry R.M., Miller D.M., Treisman J., Gailani F., Riley L., Conlon K., Pockaj B. (2011). gp100 Peptide Vaccine and Interleukin-2 in Patients with Advanced Melanoma. N. Engl. J. Med..

[95] McDermott D., Haanen J., Chen T.T., Lorigan P., O’Day S. (2013). Efficacy and safety of ipilimumab in metastatic melanoma patients surviving more than 2 years following treatment in a phase III trial (MDX010-20). Ann. Oncol..

[96] Dany M., Nganga R., Chidiac A., Hanna E., Matar S., Elston D. (2016). Advances in immunotherapy for melanoma management. Hum. Vaccines Immunother..

[97] Robbins P.D., Morelli A.E. (2014). Regulation of immune responses by extracellular vesicles. Nat. Rev. Immunol..

[98] Pitt J.M., Charrier M., Viaud S., Andre F., Besse B., Chaput N., Zitvogel L. (2014). Dendritic Cell-Derived Exosomes as Immunotherapies in the Fight against Cancer. J. Immunol..

[99] Zitvogel L., Regnault A., Lozier A., Wolfers J., Flament C., Tenza D., Ricciardi-Castagnoli P., Raposo G., Amigorena S. (1998). Eradication of established murine tumors using a novel cell-free vaccine: Dendritic cell-derived exosomes. Nat. Med..

[100] Andre F., Chaput N., Schartz N.E.C., Flament C., Aubert N., Bernard J., Lemonnier F., Raposo G., Escudier B., Hsu D.-H. (2004). Exosomes as Potent Cell-Free Peptide-Based Vaccine. I. Dendritic Cell-Derived Exosomes Transfer Functional MHC Class I/Peptide Complexes to Dendritic Cells. J. Immunol..

[101] Escudier B., Dorval T., Chaput N., André F., Caby M.P., Novault S., Flament C., Leboulaire C., Borg C., Amigorena S. (2005). Vaccination of metastatic melanoma patients with autologous dendritic cell (DC) derived-exosomes: Results of the first phase 1 clinical trial. J. Transl. Med..

[102] Paoloni M.C., Khanna C. (2007). Comparative Oncology Today. Vet. Clin. N. Am. Small Anim. Pract..

[103] Lequarré A.S., Andersson L., André C., Fredholm M., Hitte C., Leeb T., Lohi H., Lindblad-Toh K., Georges M. (2011). LUPA: A European initiative taking advantage of the canine genome architecture for unravelling complex disorders in both human and dogs. Vet. J..

[104] Simpson R.M., Bastian B.C., Michael H.T., Webster J.D., Prasad M.L., Conway C.M., Prieto V.M., Gary J.M., Goldschmidt M.H., Esplin D.G. (2014). Sporadic naturally occurring melanoma in dogs as a preclinical model for human melanoma. Pigment Cell Melanoma Res..

[105] Bergman P.J. (2007). Canine Oral Melanoma. Clin. Tech. Small Anim. Pract..

[106] Ranieri G., Gadaleta C.D., Patruno R., Zizzo N., Daidone M.G., Hansson M.G., Paradiso A., Ribatti D. (2013). A model of study for human cancer: Spontaneous occurring tumors in dogs. Biological features and translation for new anticancer therapies. Crit. Rev. Oncol. Hematol..

[107] Paoloni M., Khanna C. (2008). Translation of new cancer treatments from pet dogs to humans. Nat. Rev. Cancer.

[108] Felsburg P.J. (2002). Overview of immune system development in the dog: Comparison with humans. Hum. Exp. Toxicol..

[109] Holsapple M.P., West L.J., Landreth K.S. (2003). Species comparison of anatomical and functional immune system development. Birth Defects Res. Part B Dev. Reprod. Toxicol..

[110] Park J.S., Withers S.S., Modiano J.F., Kent M.S., Chen M., Luna J.I., Culp W.T.N., Sparger E.E., Rebhun R.B., Monjazeb A.M. (2016). Canine cancer immunotherapy studies: Linking mouse and human. J. Immunother. Cancer.

[111] Cobbold S., Metcalfe S. (1994). Monoclonal antibodies that define canine homologues of human CD antigens: Summary of the First International Canine Leukocyte Antigen Workshop (CLAW). Tissue Antigens.

[112] Isotani M., Katsuma K., Tamura K., Yamada M., Yagihara H., Azakami D., Ono K., Washizu T., Bonkobara M. (2006). Efficient generation of canine bone marrow-derived dendritic cells. J. Vet. Med. Sci..

[113] Michael H.T., Ito D., McCullar V., Zhang B., Miller J.S., Modiano J.F. (2013). Isolation and characterization of canine natural killer cells. Vet. Immunol. Immunopathol..

[114] Estrela-Lima A., Araújo M.S.S., Costa-Neto J.M., Teixeira-Carvalho A., Barrouin-Melo S.M., Cardoso S.V., Martins-Filho O.A., Serakides R., Cassali G.D. (2010). Immunophenotypic features of tumor infiltrating lymphocytes from mammary carcinomas in female dogs associated with prognostic factors and survival rates. BMC Cancer.

[115] Raposo T., Gregório H., Pires I., Prada J., Queiroga F.L. (2014). Prognostic value of tumour-associated macrophages in canine mammary tumours. Vet. Comp. Oncol..

[116] Bergeron L.M., McCandless E.E., Dunham S., Dunkle B., Zhu Y., Shelly J., Lightle S., Gonzales A., Bainbridge G. (2014). Comparative functional characterization of canine IgG subclasses. Vet. Immunol. Immunopathol..

[117] Maekawa N., Konnai S., Ikebuchi R., Okagawa T., Adachi M., Takagi S., Kagawa Y., Nakajima C., Suzuki Y., Murata S. (2014). Expression of PD-L1 on canine tumor cells and enhancement of IFN-γ production from tumor-infiltrating cells by PD-L1 blockade. PLoS ONE.

[118] Tagawa M., Maekawa N., Konnai S., Takagi S. (2016). Evaluation of costimulatory molecules in peripheral blood lymphocytes of canine patients with histiocytic sarcoma. PLoS ONE.

[119] Maekawa N., Konnai S., Takagi S., Kagawa Y., Okagawa T., Nishimori A., Ikebuchi R., Izumi Y., Deguchi T., Nakajima C. (2017). A canine chimeric monoclonal antibody targeting PD-L1 and its clinical efficacy in canine oral malignant melanoma or undifferentiated sarcoma. Sci. Rep..

[120] Nishiya A., Massoco C., Felizzola C., Perlmann E., Batschinski K., Tedardi M., Garcia J., Mendonça P., Teixeira T., Zaidan Dagli M. (2016). Comparative Aspects of Canine Melanoma. Vet. Sci..

[121] Atherton M.J., Morris J.S., McDermott M.R., Lichty B.D. (2016). Cancer immunology and canine malignant melanoma: A comparative review. Vet. Immunol. Immunopathol..

[122] Rivera R.S., Nagatsuka H., Gunduz M., Cengiz B., Gunduz E., Siar C.H., Tsujigiwa H., Tamamura R., Han K.N., Nagai N. (2008). C-kit protein expression correlated with activating mutations in KIT gene in oral mucosal melanoma. Virchows Arch..

[123] Tsao H., Chin L., Garraway L.A., Fisher D.E. (2012). Melanoma: From mutations to medicine. Genes Dev..

[124] Mochizuki H., Kennedy K., Shapiro S.G., Breen M.B. (2015). BRAF mutations in canine cancers. PLoS ONE.

[125] Chang A.E., Karnell L.H., Menck H.R. (1998). The national cancer data base report on cutaneous and noncutaneous melanoma: A summary of 84,836 cases from the past decade. Cancer.

[126] Kong Y., Si L., Zhu Y., Xu X., Corless C.L., Flaherty K.T., Li L., Li H., Sheng X., Cui C. (2011). Large-scale analysis of KIT aberrations in Chinese patients with melanoma. Clin. Cancer Res..

[127] Del Vecchio M., Di Guardo L., Ascierto P.A., Grimaldi A.M., Sileni V.C., Pigozzo J., Ferraresi V., Nuzzo C., Rinaldi G., Testori A. (2014). Efficacy and safety of ipilimumab 3 mg/kg in patients with pretreated, metastatic, mucosal melanoma. Eur. J. Cancer.

[128] Hernandez B., Adissu H.A., Wei B.R., Michael H.T., Merlino G., Mark Simpson R. (2018). Naturally occurring canine melanoma as a predictive comparative oncology model for human mucosal and other triple wild-type melanomas. Int. J. Mol. Sci..

[129] Boston S.E., Lu X., Culp W.T.N., Montinaro V., Romanelli G., Dudley R.M., Liptak J.M., Mestrinho L.A., Buracco P. (2014). Efficacy of systemic adjuvant therapies administered to dogs after excision of oral malignant melanomas: 151 cases (2001–2012). J. Am. Vet. Med. Assoc..

[130] Brockley L.K., Cooper M.A., Bennett P.F. (2013). Malignant melanoma in 63 dogs (2001–2011): The effect of carboplatin chemotherapy on survival. N. Z. Vet. J..

[131] Quintin-Colonna F., Devauchelle P., Fradelizi D., Mourot B., Faure T., Kourilsky P., Roth C., Mehtali M. (1996). Gene therapy of spontaneous canine melanoma and feline fibrosarcoma by intratumoral administration of histoincompatible cells expressing human interleukin-2. Gene Ther..

[132] Hogge G.S., Burkholder J.K., Culp J., Albertini M.R., Dubielzig R.R., Yang N.-S., MacEwen E.G. (1999). Preclinical development of human granulocyte-macrophage colony-stimulating factor-transfected melanoma cell vaccine using established canine cell lines and normal dogs. Cancer Gene Ther..

[133] Finocchiaro L.M.E., Fiszman G.L., Karara A.L., Glikin G.C. (2008). Suicide gene and cytokines combined nonviral gene therapy for spontaneous canine melanoma. Cancer Gene Ther..

[134] Glikin G.C., Finocchiaro L.M.E. (2014). Clinical trials of immunogene therapy for spontaneous tumors in companion animals. Sci. World J..

[135] Atkins M.B., Lotze M.T., Dutcher J.P., Fisher R.I., Weiss G., Margolin K., Abrams J., Sznol M., Parkinson D., Hawkins M. (1999). High-dose recombinant interleukin 2 therapy for patients with metastatic melanoma: Analysis of 270 patients treated between 1985 and 1993. J. Clin. Oncol..

[136] Kaufman H.L., Ruby C.E., Hughes T., Slingluff C.L. (2014). Current status of granulocyte-macrophage colony-stimulating factor in the immunotherapy of melanoma. J. Immunother. Cancer.

[137] Rotte A., Bhandaru M., Zhou Y., McElwee K.J. (2015). Immunotherapy of melanoma: Present options and future promises. Cancer Metastasis Rev..

[138] Bergman P.J., McKnight J., Novosad A., Charney S., Farrelly J., Craft D., Wulderk M., Jeffers Y., Sadelain M., Hohenhaus A.E. (2003). Long-term survival of dogs with advanced malignant melanoma after DNA vaccination with xenogeneic human tyrosinase: A phase I trial. Clin. Cancer Res..

[139] Liao J.C.F., Gregor P., Wolchok J.D., Orlandi F., Craft D., Leung C., Houghton A.N., Bergman P.J. (2006). Vaccination with human tyrosinase DNA induces antibody responses in dogs with advanced melanoma. Cancer Immun..

[140] Grosenbaugh D.A., Leard A.T., Bergman P.J., Klein M.K., Meleo K., Susaneck S., Hess P.R., Jankowski M.K., Jones P.D., Leibman N.F. (2011). Safety and efficacy of a xenogeneic DNA vaccine encoding for human tyrosinase as adjunctive treatment for oral malignant melanoma in dogs following surgical excision of the primary tumor. Am. J. Vet. Res..

[141] Ottnod J.M., Smedley R.C., Walshaw R., Hauptman J.G., Kiupel M., Obradovich J.E. (2013). A retrospective analysis of the efficacy of Oncept vaccine for the adjunct treatment of canine oral malignant melanoma. Vet. Comp. Oncol..

[142] McLean J.L., Lobetti R.G. (2015). Use of the melanoma vaccine in 38 dogs: The South African experience. J. S. Afr. Vet. Assoc..

[143] Bergman P.J., Camps-Palau M.A., McKnight J.A., Leibman N.F., Craft D.M., Leung C., Liao J., Riviere I., Sadelain M., Hohenhaus A.E. (2006). Development of a xenogeneic DNA vaccine program for canine malignant melanoma at the Animal Medical Center. Vaccine.

[144] Wolchok J.D., Yuan J., Houghton A.N., Gallardo H.F., Rasalan T.S., Wang J., Zhang Y., Ranganathan R., Chapman P.B., Krown S.E. (2007). Safety and immunogenicity of tyrosinase DNA vaccines in patients with melanoma. Mol. Ther..

[145] Yuan J., Ku G.Y., Adamow M., Mu Z., Tandon S., Hannaman D., Chapman P., Schwartz G., Carvajal R., Panageas K.S. (2013). Immunologic responses to xenogeneic tyrosinase DNA vaccine administered by electroporation in patients with malignant melanoma. J. Immunother. Cancer.

[146] Treggiari E., Grant J.P., North S.M. (2016). A retrospective review of outcome and survival following surgery and adjuvant xenogeneic DNA vaccination in 32 dogs with oral malignant melanoma. J. Vet. Med. Sci..

[147] Denies S., Cicchelero L., Polis I., Sanders N.N. (2016). Immunogenicity and safety of xenogeneic vascular endothelial growth factor receptor-2 DNA vaccination in mice and dogs. Oncotarget.

[148] Piras L.A., Riccardo F., Iussich S., Maniscalco L., Gattino F., Martano M., Morello E., Lorda Mayayo S., Rolih V., Garavaglia F. (2016). Prolongation of survival of dogs with oral malignant melanoma treated by en bloc surgical resection and adjuvant CSPG4-antigen electrovaccination. Vet. Comp. Oncol..

[149] Riccardo F., Iussich S., Maniscalco L., Mayayo S.L., La Rosa G., Arigoni M., De Maria R., Gattino F., Lanzardo S., Lardone E. (2014). CSPG4-specific immunity and survival prolongation in dogs with oral malignant melanoma immunized with human CSPG4 DNA. Clin. Cancer Res..

[150] Price M.A., Colvin Wanshura L.E., Yang J., Carlson J., Xiang B., Li G., Ferrone S., Dudek A.Z., Turley E.A., McCarthy J.B. (2011). CSPG4, a potential therapeutic target, facilitates malignant progression of melanoma. Pigment Cell Melanoma Res..

[151] Rolih V., Barutello G., Iussich S., De Maria R., Quaglino E., Buracco P., Cavallo F., Riccardo F. (2017). CSPG4: A prototype oncoantigen for translational immunotherapy studies. J. Transl. Med..

[152] Mayayo S.L., Prestigio S., Maniscalco L., La Rosa G., Aricò A., De Maria R., Cavallo F., Ferrone S., Buracco P., Iussich S. (2011). Chondroitin sulfate proteoglycan-4: A biomarker and a potential immunotherapeutic target for canine malignant melanoma. Vet. J..

[153] Yang V.K., Loughran K.A., Meola D.M., Juhr C.M., Thane K.E., Davis A.M., Hoffman A.M. (2017). Circulating exosome microRNA associated with heart failure secondary to myxomatous mitral valve disease in a naturally occurring canine model. J. Extracell. Vesicles.

[154] Brandt L.E., Ehrhart E.J., Scherman H., Olver C.S., Bohn A.A., Prenni J.E. (2014). Characterization of the canine urinary proteome. Vet. Clin. Pathol..

